# GLP-1 receptor agonists beyond diabetes management

**DOI:** 10.1016/j.eclinm.2024.103021

**Published:** 2024-12-17

**Authors:** eClinicalMedicine

The first glucagon-like peptide-1 receptor agonist (GLP-1 RA], exenatide, was approved by US Food and Drug Administration in 2005, and was initially indicated for the treatment of type 2 diabetes. Exenatide is a synthetic version of exendin-4, a hormone found in the saliva of a lizard native to North America that can survive extended periods without food. Exendin-4 can mimic the action of GLP-1, an incretin regulating glucose homeostasis. GLP-1 acts on pancreatic β cells to enhance insulin secretion and reduce glucagon secretion, slows gastric emptying, and promotes satiety, ultimately reducing appetite and decreasing energy intake.

Since 2005, the landscape of available GLP-1 RAs has greatly expanded alongside the number of their therapeutic indications. Beyond exenatide, the class now includes liraglutide, dulaglutide, lixisenatide, tirzepatide, and semaglutide (marketed as Ozempic or Wegovy). Both liraglutide and dulaglutide have shown cardiovascular risk reduction in landmark trials (LEADER and REWIND, respectively), underscoring their dual benefits for glucose homeostasis and cardiovascular health. Semaglutide is highly effective for weight loss and has cardiovascular benefits as evidenced in SUSTAIN trials. Most recently, tizepatide, a dual GLP-1 and glucose-dependent insulinotropic polypeptide receptor agonist, has shown superior glycaemic control compared with the available single agonist agents, setting a new standard for diabetes and weight management.

GLP-1 RAs have revolutionised the field of diabetes management and metabolic control, given their pronounced advantages over earlier anti-diabetic therapies. Their ability to promote weight loss makes them particularly beneficial for managing type 2 diabetes, which often coexists with obesity. Additionally, their cardiovascular benefits further enhance their value in addressing the broader health risks associated with diabetes.

The lists of beneficial effects for these agonists keep growing. In November, 2024, during the American Association for the Study of Liver Disease conference in San Diego, CA, investigators of the ongoing ESSENCE trial, which is testing the effect of semaglutide in metabolic dysfunction-associated steatohepatitis (MASH), presented promising results of their interim analysis. Semaglutide showed superiority over placebo in achieving the two primary endpoints: resolution of steatohepatitis without worsening fibrosis, and improvement in liver fibrosis without exacerbation of steatohepatitis. In other trials, semaglutide also reduced the risk of kidney disease progression and kidney-related deaths. Liraglutide improved ovarian dysfunction in women with polycystic ovary syndrome who had overweight. Observational studies have also linked the use of semaglutide with a reduced risk of dementia and other neurological disorders, as well as addiction behaviours (nicotine and alcohol consumption) in people with type 2 diabetes.

The ever-increasing list of pathologies potentially improved by these agonists raises the question of how we prioritise those who will benefit most. The benefits of GLP-1 RAs now extend far beyond diabetes management. For example, GLP-1 RAs, such as liraglutide and semaglutide, are approved for obesity treatment in individuals without diabetes. Their introduction has catalysed a positive paradigm shift in the management of obesity, redefining it as a treatable, chronic medical condition rather than a consequence of lifestyle choices. Despite GLP-1 RAs’ proven efficacy in treating diabetes, obesity, and other cardiometabolic disease, their use is not without concerns and barriers. Some concerns relate to the adverse effects associated with their use, with gastrointestinal reactions, including nausea, vomiting, diarrhoea, and constipation being the most common, particularly in the early stages of treatment. Although these reactions are generally mild and transient, they can occasionally lead to severe complications such as dehydration and, if not managed effectively, they can lead to hospitalisation. Other serious but less frequent concerns include the potential for acute pancreatitis and gallbladder disease.

The off-label use of GLP-1 RAs further complicates the quantification of their adverse effects. Clinical trials for these drugs have typically included patients with diabetes or obesity, or both, **which** leaves a gap in robust safety data for people using them off-label. In addition, because these medications are increasingly used for aesthetic or performance-driven purposes, adverse events might be underreported, particularly when usage occurs without medical supervision.

The increasing use of GLP-1 RAs in individuals who have neither diabetes nor obesity poses concerns regarding accessibility. Newer agents, such as semaglutide and tirzepatide, are generally expensive, even if the costs vary substantially between countries. The high demand for and limited supply of GLP-1 RAs, caused in part by off-label use, creates barriers to access for patients who are eligible to receive treatment with these drugs according to medical or regulatory guidelines, which can lead to negative health consequences for these individuals. Limited availability and high costs have driven patients to explore alternative means of obtaining these medications and this fuelled alternative markets, including online clinics, often unauthorised, that offer GLP-1 RA or compounded medications of unknown origin at discounted prices. The costs are even more prohibitive in low-income and middle-income countries due to weaker health-care subsidies, import taxes, and scarcity of local production facilities, making GLP-1 RAs largely inaccessible to most of the population.

The potential of GLP-1 RAs is undeniable, with their benefits on diabetes and obesity management already redefining care. Emerging evidence, including data from patients with MASH and kidney diseases, will only continue to expand the list of indications for this class of drugs. However, realising this promise requires important barriers to be addressed. Accessibility barriers, driven by excessive costs and supply constraints, must be tackled to reduce disparities, especially in low-resource settings. Effective regulation and prioritisation are essential to ensure that those patients most likely to benefit receive the treatment, and to minimise misuse and associated harms. Educating patient and health-care provider is equally essential to ensure informed decision making, improve adherence, and tackle stigma. Stakeholders, including researchers, health-care providers, patients and advocacy groups, policy makers and pharmaceutical companies, must strike a balance between innovation, public health priorities, and equitable access. Global strategies, such as differential pricing, local manufacturing, subsidised programmes, and transparent policies, should make these transformative therapies more accessible and affordable in all socioeconomic contexts.

GLP-1 RAs have truly been game changers in medicine, but their full potential can only be achieved through rigorous clinical trials and a collaborative focus on equity, education, and sustainable integration into health-care systems worldwide.

